# Attenuation of *miR-34a* protects cardiomyocytes against hypoxic stress through maintenance of glycolysis

**DOI:** 10.1042/BSR20170925

**Published:** 2017-11-06

**Authors:** Ying Zhang, Gang Liu, Xiaogang Gao

**Affiliations:** Department of Internal Medicine, Tianjin Huanhu Hospital, 6 Jinzhao Road, Jinnan District, Tianjin, China, 300060

**Keywords:** cardiomyocytes dysfunction, glycolysis, hypoxic stress, LDHA, miR-34a

## Abstract

MiRNAs are a class of endogenous, short, single-stranded, non-coding RNAs, which are tightly linked to cardiac disorders such as myocardial ischemia/reperfusion (I/R) injury. *MiR-34a* is known to be involved in the hypoxia-induced cardiomyocytes apoptosis. However, the molecular mechanisms are unclear. In the present study, we demonstrate that under low glucose supply, rat cardiomyocytes are susceptible to hypoxia. Under short-time hypoxia, cellular glucose uptake and lactate product are induced but under long-time hypoxia, the cellular glucose metabolism is suppressed. Interestingly, an adaptive up-regulation of *miR-34a* by long-time hypoxia was observed both *in vitro* and *in vivo*, leading to suppression of glycolysis in cardiomyocytes. We identified lactate dehydrogenase-A (LDHA) as a direct target of *miR-34a*, which binds to the 3′-UTR region of *LDHA* mRNA in cardiomyocytes. Moreover, inhibition of *miR-34a* attenuated hypoxia-induced cardiomyocytes dysfunction through restoration of glycolysis. The present study illustrates roles of *miR-34a* in the hypoxia-induced cardiomyocytes dysfunction and proposes restoration of glycolysis of dysfunctional cardiomyocytes by inhibiting *miR-34a* during I/R might be an effectively therapeutic approach against I/R injury.

## Introduction

During myocardial infarction, ischemia and/or reperfusion is the major cause of cardiomyocyte dysfunction, leading to worldwide morbidity and mortality [1]. Under physiological conditions, sufficient supply of oxygen is indispensable for maintenance of cardiac viability and function [2]. During ischemia-associated hypoxia, a series of abrupt biochemical and metabolic changes within the myocardium occur due to the deprivation of oxygen and nutrient supply [3]. Low oxygen halts oxidative phosphorylation, leading to mitochondrial dysfunction, ATP depletion, and inhibition of myocardial contractile function [4].

MiRNAs are a group of short non-coding RNAs (~20–25 nts in length) which down-regulate mRNAs of their target genes expression through binding to 3′-UTR [5]. Moreover, through modulating mRNA expressions, miRNAs are known to involve in regulating cell proliferation, differentiation, apoptosis, autophagy, and development [6,7]. Recent publications reported that miRNAs play important roles during myocardial infarction [8], ischemia/reperfusion (I/R) [9], and arrhythmia [10]. A recent study revealed that under cardiomyocyte hypoxia/reoxygenation (H/R) injury, alterations in miRNAs expression occur, leading to disturbances in downstream apoptotic pathway regulation [11]. Moreover, Sarkar et al. reported that by targetting the programmed cell death 4 (*PDCD4*) gene, *miR-21* inhibits cell death under H/R conditions [12]. It been known that down-regulation of *miR-34a* could reduce myocardial I/R injury by inhibiting cardiomyocyte apoptosis through targetting BCL-2 [13], suggesting a protective role of *miR-34a* during heart ischemia stress. Furthermore, several miRNAs, such as *miR-199a* and *miR-214* [14], *miR-494* [15], *miR-499* [16], and *miR-24* [17] are known to protect cells from hypoxia- or ischemia-induced damage.

Glycolysis is the biochemical process that converts glucose into lactate or pyruvate under anaerobic or aerobic conditions with net production of 2 moles of ATP [18]. It is known that during ischemia, glycolysis becomes a very important source of energy due to its ability to generate ATP [19]. In the present study, we will investigate the roles of *miR-34a* in hypoxia-induced cardiomyocytes death. In addition, the potential targets of *miR-34a* in the glycolysis pathway will be identified.

## Materials and methods

### Cell culture and low oxygen treatment

The H9c2 rat heart derived cell line obtained from the cell bank of the Chinese Academy of Sciences was cultured in Dulbecco’s modified Eagle’s medium (DMEM, Celbio) supplemented with 10% heat-inactivated FBS. Hypoxia was induced by exposing cells to 1% O_2_, 94% N_2_, and 5% CO_2_ for 12, 24, 48, or 72 h using a modular incubator (Model 3131, Forma Scientific, Marietta, OH, U.S.A.). Cells cultured under a normoxic atmosphere served as the control.

### Rat hypoxia model

The hypoxic environment for rat was created in a hypoxia chamber according to a previous report [20]. O_2_ cabinets with manual purge airlock were installed with glove boxes, oxygen control system with an oxygen sensor, a nitrogen and oxygen gas regulator, gloveless sleeves and arm port plugs, an internal circulation fan, and compact dehumidifier. Age-matched (10-week-old) male Sprague–Dawley rats (weighing 270–320 g) were exposed to the hypoxic or normoxic environment. For hypoxic environment, oxygen concentration was gradually decreased from normoxia (20.9%) to 7% (1% down per day) within 2 weeks followed by an additional 3 or 14 days hypoxia exposure at 7% O_2_ to avoid hypobaropathy caused by a rapid drop in partial oxygen pressure. Rats were killed and hearts were harvested for cardiomyocyte isolation at the end of the hypoxia experiment.

### Antibodies and reagents

Rabbit monoclonal lactate dehydrogenase-A (LDHA) antibody was ordered from Cell Signaling (#2012); mouse monoclonal antibody against Hexokinase 2 was purchased from Santa Cruz (sc-6521); mouse monoclonal antibody against β-actin was purchased from Santa Cruz Biotechnology (sc-47778). Oxamate was purchased from Sigma–Aldrich (St. Louis, MO, U.S.A.). *MiR-34a* mimic, *miR-34a* inhibitor, and their corresponding negative control were purchased from Shanghai GenePharma Co. Ltd. (Shanghai, China).

### Transfection of miRNAs and siRNA

*MiR-34a* mimic, inhibitor, or control miRNAs were transfected into H9c2 cells at a concentration of 50 nM for 48 h using Lipofectamine 3000 (Invitrogen, CA, U.S.A.) following the manufacturer’s instructions. LDHA siRNA or control siRNA was transfected into H9c2 cells at a concentration of 100 nM for 72 h using Lipofectamine 3000 (Invitrogen, CA, U.S.A.) following the manufacturer’s instructions.

### Luciferase reporter assay

The wild-type or mutant 3′-UTR sequences of LDHA were synthesized and subcloned into a PsiCheck2 vector. Cells were cotransfected with 100 ng plasmids containing wild-type or mutant 3′-UTR of LDHA with 50 nM *miR-34a* mimic or control miRNAs for 48 h according to manufacturer’s instructions using Lipofectamine 2000 reagent (Thermo Fisher Scientific). After transfection, luciferase activities were measured using the Luciferase Dual-Reporter Kit (Promega) according to manufacturer’s instructions. Experiments were performed in triplicate.

### Real-time quantitative PCR

The expressions of *miR-34a* were examined by real-time quantitative PCR (qPCR). Total RNA was extracted from H9c2 cells using the TRIzol reagent (Invitrogen Life Technologies). A poly-A tail was added to the extracted total RNA, which was then reverse transcribed into cDNA using the qScript™ microRNA cDNA Synthesis Kit from Quanta Biosciences (Beverly, MA, U.S.A.) according to the manufacturer’s instructions. RT-qPCR was conducted using the TaqMan microRNA assays kit (Applied Biosystems) according the manufacturer’s instructions. All reactions were performed in triplicate. Human U6 served as an internal control. The relative expressions of miRNAs were calculated using the comparative *C*_T_ method (2^−ΔΔ*C*^_t_). Experiments were performed in triplicate.

### Measurements of glucose uptake and lactate production

The glucose uptake of H9c2 cells was measured using the Glucose Uptake Assay Kit (Colorimetric) from Abcam (ab136955) according to the manufacturer’s instructions. The lactate product was measured using the L-Lactate Assay Kit (Colorimetric) from Abcam (ab65331) according to the manufacturer’s instructions. The results were normalized by protein concentration of each treatment. Experiments were performed in triplicate.

### Oxygen consumption rate

The oxygen consumption rate (OCR) of H9c2 cells was measured using the Extracellular Oxygen Consumption Assay from Abcam (ab197243) according to the manufacturer’s instructions. The results were normalized by protein concentration of each treatment. Experiments were performed in triplicate.

### Detection of cell viability

The cells viability was tested by MTT assay (Sigma–Aldrich). Briefly, equal number of cells were plated into 96-well plate for overnight. Following incubation and treatment with hypoxia or control, the MTT reagent was added into cell culture medium at a concentration of 0.5 mg/ml at 37°C for 4 h. DMSO was then added into each well to dissolve formazan crystals. The absorbance was measured on an Optimax Microplate Reader (Molecular Devices, Sunnyvale, CA) at a wavelength of 570 nm with background subtracted at 690 nm. Experiments were performed in triplicate.

### Immunohistochemical staining

The cardiac tissue was isolated from rat and after fixation and sectioning, slides were stained by primary antibodies against Hexokinase 2 and LDHA to assess the protein expressions. Slides were washed by PBS and incubated with secondary antibody. Images were captured using a microscope (BX51; Olympus Corporation, Tokyo, Japan).

### Western blot analysis

Total protein in cells from each treatment were extracted by lysis buffer (20 mM HEPES, pH 7.0, 5 mM DTT, 2 mM EDTA, 0.1% 3-[(3 cholaminodopropyl)dimethylammonio]-1-propanesulphonate, 0.1% Triton X-100, 1 mM phenylmethylsulphonyl fluoride, 1 Wg/ml each aprotinin, pepstatin, and leupeptin). Protein concentrations were measured by Bradford assay. Samples were diluted to 1:1 with Laemmli buffer, then desaturated by boiling for 5 min. Equal amount protein of each sample was loaded into SDS/PAGE (10% gel) followed by transfer to PVDF membrane. The membrane was blocked by 5% BSA at room temperature for 1 h and incubated with primary antibodies at 4°C for overnight. After washing, the membrane was incubated for 30 min with the secondary antibody (horseradish peroxidase conjugated). After washing, immunoreactive bands were visualized with an ECL kit (Pierce). Quantitative analysis of immunoblotted bands was performed by ImageJ software. Experiments were performed in triplicate.

### Statistical analysis

Statistical analysis was performed using Prism 5.0 software. All data are expressed as the mean ± standard deviation (STD), Student’s *t* test was used to compare the difference between two groups and *P*<0.05 was considered statistically significant.

## Results

### Low glucose and oxygen triggers cardiomyocytes death

**Figure 1:**
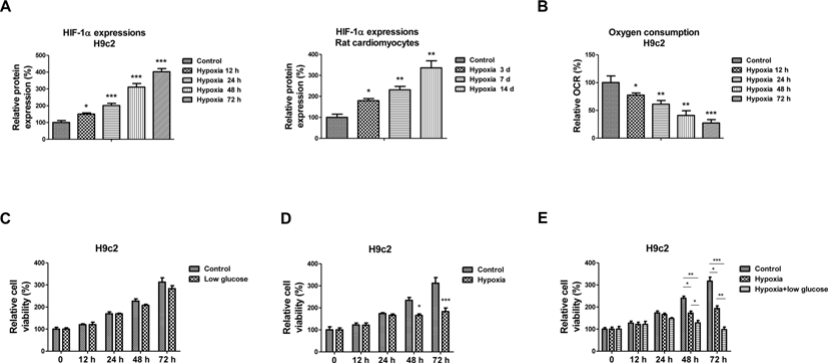
Cardiomyocytes are more sensitive to hypoxia under low glucose condition (**A**) Rat cardiac cell line, H9c2 (left) was treated with hypoxia for 0, 12, 24, 48, or 72 h. The expressions of Hif-1α were measured by Western blot. The expressions of Hif-1α from rat primary cardiomyocytes isolated from hypoxia-treated rats (right) were analyzed by Western blot. β-actin was the internal control. The relative expressions of Hif-1 α were quantitated using ImageJ software. (**B**) H9c2 cells were treated with hypoxia for 0, 12, 24, 48, or 72 h. Oxygen consumption was analyzed. (**C**) H9c2 cells were treated with or without low glucose condition for 0, 12, 24, 48, or 72 h, cell viability was analyzed by MTT assay. (**D**) H9c2 cells were treated with or without low oxygen condition for 0, 12, 24, 48 or 72 h, cell viability was analyzed by MTT assay. (**E**) H9c2 cells were treated with normoxia, hypoxia alone or low glucose plus low oxygen conditions for 0, 12, 24, 48, or 72 h, cell viability was analyzed by MTT assay. Data are shown as the means ± S.E.M.; *P<0.05; **P<0.01; ***P<0.001.

Hypoxia is a major factor for the ischemia and/or reperfusion induced cardiomyocyte apoptosis during myocardial infarction [21]. Moreover, it has been widely studied that cellular nutrition metabolism plays essential roles during IR of cardiomyocytes [19]. We started to test the effects of hypoxia and/or low glucose on the cardiomyocytes survival. Rat cardiomyocytes H9c2 were exposed to hypoxia for 12, 24, 48, and 72 h and found that the Hif-1α expression, which is a marker of cellular response to hypoxia, was significantly induced by hypoxia (Figure 1A left). Moreover, we performed *in vivo* experiments using a rat hypoxic model. We gradually dropped the oxygen by 1% per day from 20.9% (regular room air oxygen) to 7% within 2 weeks followed by exposure to 7% oxygen for an additional 3 or 14 days to avoid hypobaropathy caused by a rapid drop in partial oxygen pressure. Rats were then killed and cardiomyocytes were isolated from heart. Consistently, our results demonstrated a significant induction in Hif-1α expression (Figure 1A right). Oxygen consumption was found significantly decreased under hypoxia at 12, 24, 48, or 72 h by 77, 61, 40, or 27%, respectively (Figure 1B). Results in Figure 1C showed H9c2 cells under low glucose condition did not display significant cell death. In addition, exposure to short time (12/24 h) hypoxia did not induce H9c2 cell death. Cell viability was suppressed by low oxygen condition at 48 and 72 h from 234 to 165% and 311 to 183%, respectively (Figure 1D). However, with oxygen deprivation, cells cultured in low glucose supply medium at 48 and 72 h significantly exacerbated cardiomyocytes viability compared with control and hypoxia only condition (Figure 1E). These results demonstrated during hypoxia, glucose metabolism is essential for the survival of cardiomyocytes.

### Short-time hypoxia induces glycolysis but long-time hypoxia suppresses glycolysis in cardiomyocytes

**Figure 2:**
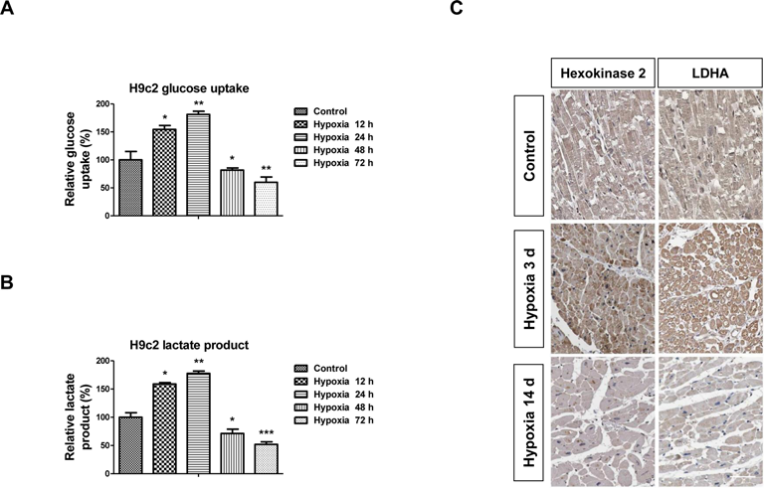
Glucose metabolism in H9c2 cells are altered under hypoxic conditions H9c2 cells were treated with low oxygen condition for 0, 12, 24, 48, or 72 h, the glucose uptake (**A**) and lactate product (**B**) were measured. (**C**) IHC staining of Hexokinase 2 and LDHA in rat heart tissues under 0, 3, or 14 days’ hypoxia treatments. Data are shown as the means ± S.E.M.; *P<0.05; **P<0.01; ***P<0.001. IHC; immunohistochemistry

We next investigated the effects of hypoxia on the cellular metabolism. Based on the above results (Figure 1), we hypothesized that the anaerobic glycolysis in cardiomyocytes will be affected by hypoxia. We measured the two glucose metabolism processes, glucose uptake, which detected the overall glucose utilization and lactate product, which detected the conversion of pyruvate to lactate but not acetyl-CoA [22]. As we expected, H9c2 cells with hypoxia treatments showed increased glucose uptake (Figure 2A) and lactate (Figure 2B) at 12 and 24 h exposure. However, we found that long-time hypoxia exposure suppressed glycolysis in H9c2 cells. The glucose uptake (Figure 2A) and lactate (Figure 2B) in H9c2 cells were significantly decreased at 48 and 72 h hypoxia exposure: glucose uptake was decreased to 81% (48 h) and 59% (72 h); lactate product was decreased to 71% (48 h) and 52% (72 h). Consistently, immunohistochemistry results demonstrated that the glycolysis speed-limited enzymes, Hexokinase 2 and LDHA expressions were up-regulated in rat cardiomyocytes isolated from rat with short (3 days) but inhibited by long-time hypoxic exposure (14 days) (Figure 2C). These results suggest an adaptive up-regulation of glycolysis under hypoxia may that contribute to the improvements of cardiomyocytes functions during acute ischemia but impair cardiomyocytes viability through decreased glycolysis under long-time hypoxia exposure.

### Adaptive up-regulation of *miR-34a* by hypoxia

**Figure 3:**
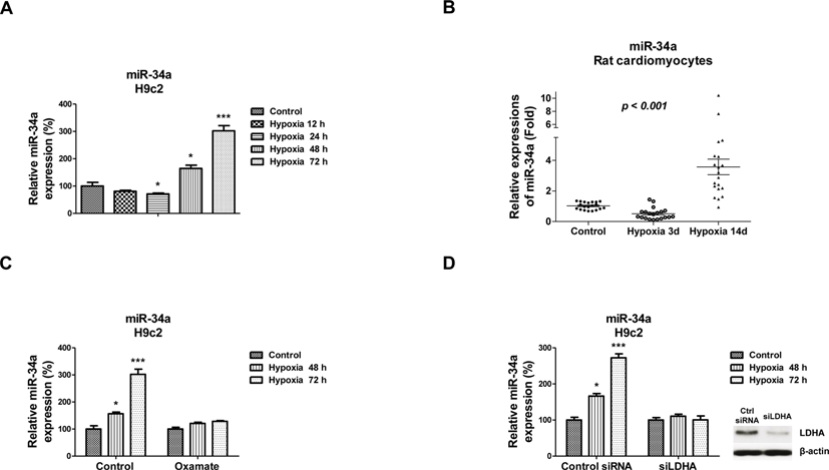
*MiR-34a* is adaptively regulated by hypoxia (**A**) H9c2 cells were treated with low oxygen conditions for 0, 12, 24, 48, or 72 h, the expressions of *miR-34a* were measured by qRT-PCR. (**B**) Rats were exposed to hypoxia using hypoxia chamber for 0, 3, or 14 days. Primary rat cardiomyocytes were isolated for measurement of *miR-34a* expressions by qRT-PCR. (**C**) H9c2 cells were treated with Oxamate for 48 h, followed by treatments with low oxygen conditions for 0, 48, or 72 h, the expressions of *miR-34a* were measured by qRT-PCR. (**D**) H9c2 cells were transfected with control siRNA or siLDHA for 48 h, followed by the treatments with low oxygen condition for 0, 48, or 72 h, the expressions of *miR-34a* were measured by qRT-PCR. The expressions of LDHA were measured by Western blot. U6 and β-actin was used as internal controls. Data are shown as the means ± S.E.M.; *P<0.05; **P<0.01; ***P<0.001.

The above results demonstrated that the hypoxia-modulated glycolysis in cardiomyocytes, to investigate the molecular mechanism. We measured the miRNAs expressions regulated by hypoxia. Amongst the miRNAs we screened, *miR-34a* was found to be inhibited by short hypoxia exposure to 71% but induced by long hypoxia at 48 and 72 h to 181 and 302%, respectively (Figure 3A). Consistently, *miR-34a* was up-regulated in long-time hypoxia exposure (14 days) in rat cardiomyocytes (Figure 3B), suggesting that the hypoxia-mediated up-regulation of *miR-34a* might contribute to the cardiomyocyte cell death. It has been reported that *miR-34a* could repress cellular glycolysis [23]. According to our results, we hypothesized that the long-time hypoxia exposure induced *miR-34a* may be due to an adaptive response to increased glycolysis, which contributes to the survival of cardiomyocytes under hypoxia. To test, we treated H9c2 cells with or without the glycolysis inhibitor, Oxamate. Then cells were exposed to hypoxia for 48 or 72 h. As we expected, suppression of glycolysis by Oxamate contradicted the up-regulation of *miR-34a* (Figure 3C). In addition, H9c2 cells were transfected with control siRNA or siLDHA for 48 h. Cells were then exposed to hypoxia for 48 or 72 h. Consistent results demonstrated that *miR-34a* was not up-regulated under hypoxia in H9c2 cells with inhibition of glycolysis by siLDHA (Figure 3D). These results revealed that *miR-34a* is adaptively regulated under hypoxia, suggesting that *miR-34a* might be a therapeutic target for improvement of cardiomyocyte functions during ischemia.

### *MiR-34a* inhibits anaerobic glycolysis in cardiomyocytes

**Figure 4:**
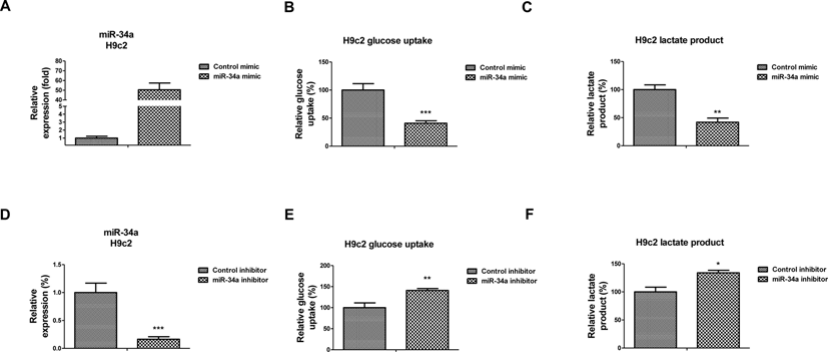
*MiR-34a* suppresses glucose metabolism in cardiomyocytes (**A**) H9c2 cells were transfected with control mimic or *miR-34a* mimic for 48 h, the expressions of *miR-34a* were measured by qRT-PCR. (**B**) H9c2 cells were transfected with control mimic or *miR-34a* mimic for 48 h, the glucose uptake and (**C**) lactate product were measured. (**D**) H9c2 cells were transfected with control inhibitor or *miR-34a* inhibitor for 48 h, the expressions of *miR-34a* were measured by qRT-PCR. (**E**) H9c2 cells were transfected with control inhibitor or *miR-34a* inhibitor for 48 h, the glucose uptake and (**F**) lactate product were measured. Data are shown as the means ± S.E.M.; *P<0.05; **P<0.01; ***P<0.001.

To investigate the roles of *miR-34a* in the hypoxia-modulated glycolysis, we transfected *miR-34a* mimics or control mimics into cardiac cell line, H9c2 (Figure 4A). The anaerobic glycolysis readouts, glucose uptake, and lactate production were measured. As we expected, overexpression of *miR-34a* in H9c2 cells significantly suppressed glucose uptake to 40% (Figure 4B) and lactate production to 42% (Figure 4C). To test whether inhibition of endogenous *miR-34a* could promote the glucose metabolism, we transfected *miR-34a* inhibitor or control inhibitor into H9c2 cells (Figure 4D). Consistently, inhibition of *miR-34a* promoted glucose uptake (Figure 4E) to 144% and lactate production to 134% (Figure 4F). Taken together, the above results demonstrated that *miR-34a* acts as a glycolysis suppressor in rat cardiomyocytes.

### *MiR-34a* directly targets LDHA in cardiomyocytes

**Figure 5:**
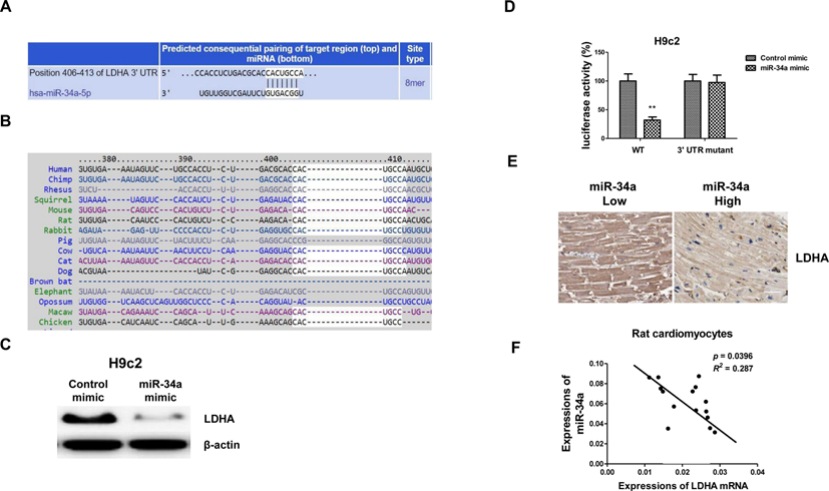
*MiR-34a* directly targets LDHA in cardiomyocytes (**A**) Potential *miR-34a* target was predicted from TargetScan.org. The position 406–413 of LDHA 3′-UTR contains putative binding sites for *miR-34a*. (**B**) Conserved *miR-34a* binding sites on LDHA 3′-UTR in multiple species. (**C**) H9c2 cells were transfected with 50 nM control mimic or *miR-34a* mimic for 48 h. Cell lysates were prepared for Western blotting analysis. β-actin was used as a loading control. (**D**) H9c2 cells were cotransfected with control mimic or *miR-34a* mimic with luciferase reporter plasmids with wild-type 3′-UTR or mutant 3′-UTR of LDHA using Lipofectamine 2000 reagent for 48 h. Cells were harvested and the luciferase activities were measured by a dual luciferase reporter assay. Results are expressed as relative LUC activity (firefly LUC/*Renilla* LUC). (**E**) IHC staining of LDHA in rat heart tissues with low or high *miR-34a* expression. (**F**) The correlation analysis of *LDHA* mRNAs and *miR-34a* from 15 rat heart tissues. Experiments were performed in triplicate. Data are shown as the means ± S.E.M.; **P<0.01.

To further examine the molecular mechanisms for the *miR-34a*-modulated glycolysis suppression, the potential targets of *miR-34a* were investigated. By searching the public miRNA database TargetScan, we found the 3′-UTR of LDHA contains a highly conserved binding site for miR-34a (Figure 5A,B). To the best of our knowledge, it has not been reported that LDHA is a direct target of *miR-34a* in cardiomyocytes. To experimentally demonstrate whether *miR-34a* targets LDHA in cardiomyocytes, *miR-34a* mimic or control mimic was transfected into H9c2 cells. Western blot results showed overexpression of *miR-34a* significantly down-regulated LDHA expressions (Figure 5C). To determine whether LDHA is a direct target of *miR-34a*, we performed a luciferase reporter analysis by cotransfecting with *miR-34a* mimic or control miRNA with a vector containing reporter-luciferase fused with either the wild-type 3′-UTR sequence or a sequence with a mutation in the predicted binding site of the 3′-UTR of *LDHA* mRNA. Cotransfection of *miR-34a* decreased the luciferase activity of the reporter containing the wild-type 3′-UTR of LDHA to 32% in H9c2 cells (Figure 5D). However, we did not detect decreased luciferase activity of the reporter fused with the mutant 3′-UTR of LDHA (Figure 5D). Moreover, we observed an invert correlation between *miR-34a* and LDHA in rat primary cardiomyocytes, low *miR-34a* expressing heart muscle tissues displayed high *LDHA* mRNA expressions (Figure 5E,F). These results demonstrate that LDHA is a direct target of *miR-34a* in cardiomyocytes.

### Attenuation of the hypoxia-induced *miR-34a* protects cardiomyocytes through restoration of LDHA

**Figure 6:**
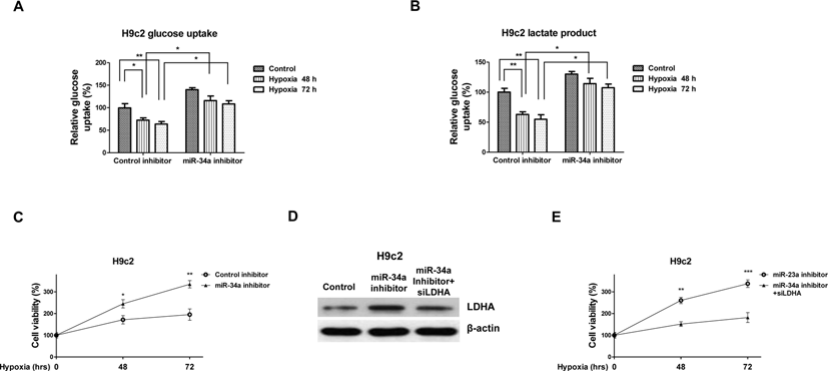
Inhibition of *miR-34a* attenuates the hypoxia-induced cardiomyocytes dysfunction through restoration of glucose metabolism (**A**) H9c2 cells were transfected with control inhibitor or *miR-34a* inhibitor for 48 h, cells were treated with hypoxia at 0, 48, or 72 h. The glucose uptake, (**B**) lactate product, and (**C**) cell viability were measured. (**D**) H9c2 cells were transfected with control siRNA, *miR-34a* inhibitor, or *miR-34a* inhibitor plus siLDHA for 48 h, followed by the detection of LDHA expression by Western blot. β-actin was the loading control. (**E**) H9c2 cells were transfected with *miR-34a* inhibitor or *miR-34a* inhibitor plus siLDHA for 48 h, cells were then exposed to hypoxia for 0, 48, or 72 h. Cell viability was measured by MTT assay. Data are shown as the means ± S.E.M.; *P<0.05; **P<0.01; ***P<0.001.

We demonstrated that hypoxia adaptively induced *miR-34a*, leading to the impaired cardiomyocytes survival at a later stage (Figures 1–3). To investigate whether inhibition of the hypoxia-induced *miR-34a* could protect cardiomyocytes under low oxygen conditions, we exposed H9c2 cells with or without miR-34a inhibition to hypoxic conditions. Our results in Figure 6A,B demonstrated inhibition of *miR-34a* increased glucose uptake and lactate production under 48 and 72 h hypoxia, suggesting inhibition of *miR-34a* might protect cardiomyocytes through restoration of glucose metabolism. As we expected, H9c2 cells displayed significantly increased survival rates from 195 to 355% with inhibiting *miR-34a* under 72 h hypoxia (Figure 6C). To examine whether the *miR-34a* inhibition mediated cardiomyocytes survival under hypoxia was through direct targetting LDHA, we transfected specific LDHA siRNA into the *miR-34a*-inhibited H9c2 cells (Figure 6D). Under hypoxia, low *miR-34a* expressing H9c2 cells with knocking down of LDHA showed decreased survival rate under 48 or 72 h hypoxia compared with control miRNAs inhibitor or control siRNA (Figure 6E). Taken together, the above results demonstrated inhibition of the hypoxia-induced *miR-34a* could protect cardiomyocytes through restoration of LDHA and glucose metabolism.

## Discussion

I/R injury is considered a major mechanism for heart transplant failure [21]. Therefore, understanding potential molecular mechanisms of I/R injury could benefit in the development of therapies. It has been known that miRNAs play important roles in the I/R injury of heart [24]. Moreover, miRNAs are important regulators involved in the influence of cardiomyocyte apoptosis resulting from ischemia-induced myocardial infarction. In the present study, we report the protection effects on the hypoxia induced cardiomyocytes dysfunction by attenuating *miR-34a* expression. In a recent publication, *miR-34a* was reported to be involved in the I/R-induced cardiomyocyte apoptosis [13]. They found that under hypoxia, inhibition of *miR-34a* significantly enhanced H9c2 cell viability through suppression of apoptosis by targetting Bcl-2. The roles of *miR-34a* in our reports were consistent with their discovery, suggesting that *miR-34a* is a potential drug target for treatment in myocardial I/R injury.

In contrast with the normal heart, where fatty acid and glucose metabolism are tightly regulated, metabolic switch between fatty acid β-oxidation and glucose oxidation occurs in ischemic-reperfused hearts [25]. Thus, an increased reliance on glycolysis as sources of ATP production was observed during ischemia during I/R. We reported that the cardiomyocytes exposed to short hypoxic environment exhibits increased glycolysis rate but suppressed glycolysis rate under long hypoxia, suggesting restoration of glycolysis of dysfunctional cardiomyocytes during I/R might be an effectively therapeutic approach against I/R injury.

The suppressive roles of *miR-34a* in glucose metabolism in multiple cancer types have been reported. *MiR-34a* targets LDHA in breast cancer [23], colon cancer [26], cervical cancer [27], and liver cancer [28]. Our results demonstrated that *miR-34a* could target LDHA in cardiomyocytes, which has not been reported before. Interestingly, we found that under short hypoxia, *miR-34a* was down-regulated but long hypoxia exposure significantly up-regulated *miR-34a*, which is inverse to the glycolysis rate under hypoxia, indicating inhibition of hypoxia-induced *miR-34a* could protect cardiomyocytes through recovering glycolysis. With the inhibition of glycolysis, we did not detect the up-regulation of *miR-34a* by hypoxia, suggesting an adaptive induction of *miR-34a*. However, the detailed mechanisms for the hypoxia-induced *miR-34a* are still under investigation. Furthermore, although we illustrated the binding sites of *miR-34a* on its target, LDHA was conserved in humans, rats, and other species (Figure 5B) and both *in vitro* and *in vivo* results consistently demonstrated the roles of *miR-34a* under hypoxia, the present study did not use human primary cardiomyocytes as its primary model. In fact, this *in vivo* rat hypoxia model provides some drawbacks like differences in biokinetic parameters or extrapolation of results to humans, limiting our study at the stage of transiting animal experiments to clinical application. In our future projects, we will focus on the roles of *miR-34a* in regulating cardiovascular disease using human cardiomyocytes from patients and their matched healthy cardio tissues.

In summary, we report an adaptive up-regulation of *miR-34a* by long-time hypoxia, leading to suppression of glycolysis rate in cardiomyocytes. We identified LDHA as a direct target of *miR-34a*, which binds to the 3′-UTR region of *LDHA* mRNA. Moreover, inhibition of *miR-34a* attenuated hypoxia-induced cardiomyocytes dysfunction. The present study illustrates roles of *miR-34a* in the hypoxia-induced cardiomyocytes dysfunction and provides molecular mechanisms for the regulation of glycolysis by *miR-34a*.

## Abbreviations

BCL-2: B-cell lymphoma 2
CT: threshold cycle
Hif-1α: hypoxia -inducible factor 1α
I/R: ischemia/reperfusion
LDHA: lactate dehydrogenase-A
qPCR: quantitative PCR
RT-qPCR: reverse transcription-quantitative
